# Doming Volume in Mitral Valve Prolapse: Pathophysiology, Imaging Implications and Clinical Relevance

**DOI:** 10.3390/jcdd13050186

**Published:** 2026-04-29

**Authors:** Francesco Mangini, Ilaria Dentamaro, Massimo Grimaldi, Marco Guglielmo, Andrea Igoren Guaricci, Francesco Spinelli, Francesca Musella, Sabino Iliceto, Antonio Di Monaco, Santo Dellegrottaglie, Simona Quarta, Luca Sgarra, Gianluigi Novielli, Robert W. W. Biederman, Sergio Suma, Stefania Marazia, Gaetano Citarelli, Roberto Calbi

**Affiliations:** 1 Cardiology Department, Ecclesiastical Entity Regional General Hospital “F. Miulli”, 70021 Acquaviva delle Fonti, Italy; 2 Cardiology Department, Policlinico di Bari University Hospital, 70124 Bari, Italy; 3 Cardiology Department, University Medical Center Utrecht, 3584 Utrecht, The Netherlands; 4 Radiology Department, Ecclesiastical Entity Regional General Hospital “F. Miulli”, 70021 Acquaviva delle Fonti, Italy; 5 Cardiology Department, Santa Maria delle Grazie Hospital, 80078 Pozzuoli, Italy; 6 Mater Dei Hospital Heart Center, LUM University, 70121 Bari, Italy; 7 Cardiology Department, Villa dei Fiori Accredited Hospital, 80011 Acerra, Italy; 8 Department of Cardiology, West Virginia University, Morgantown, WV 26506, USA; 9 Department of Cardiology, Medical University of South Carolina, Charleston, SC 29425, USA; 10 Department of Cardiology, Roper/Saint Francis Hospital, Charleston, SC 29403, USA; 11 Department of Cardiology, Parma University Hospital, 43126 Parma, Italy; 12 Department of Cardiology, Vito Fazzi Hospital, 73100 Lecce, Italy; 13 Department of Cardiology, San Paolo Hospital, 70123 Bari, Italy

**Keywords:** mitral valve prolapse, doming volume, echocardiography, cardiovascular magnetic resonance, multimodality imaging

## Abstract

Mitral valve prolapse represents the most common cause of primary mitral regurgitation in Western countries and has traditionally been viewed as a disorder driven by valvular incompetence and chronic volume overload. Within this paradigm, left ventricular enlargement was expected to correlate with regurgitant severity. However, patients with myxomatous bileaflet prolapse often exhibit left ventricular dilatation disproportionate to the degree of regurgitation, leading to the hypothesis of an intrinsic myocardial disease process. Cardiovascular magnetic resonance imaging has challenged this concept through the identification of doming volume, a previously unrecognized systolic blood compartment located between the mitral annular plane and the ventricular surface of prolapsing leaflets. This volume is mechanically coupled to ventricular contraction and contributes to total ventricular volume load independently of transvalvular regurgitation. Recognition of doming volume provides a physiological explanation for excessive ventricular remodeling observed in bileaflet prolapse and Barlow disease. Doming volume has important implications for imaging assessment. Its common exclusion from echocardiographic volumetric measurements may result in underestimation of left ventricular end-systolic volume, overestimation of ejection fraction, and underestimation of regurgitant burden, contributing to discordance between echocardiographic and cardiovascular magnetic resonance-derived measurements. Cardiovascular magnetic resonance enables comprehensive assessment, allowing accurate quantification of ventricular volumes, mitral regurgitation severity, doming volume, and myocardial tissue characteristics. Integration of doming volume into the evaluation of mitral valve prolapse improves physiological consistency between imaging findings and ventricular remodeling. However, further evidence is required before doming volume assessment can be incorporated into operative clinical indications or decision-making thresholds.

## 1. Background

Mitral valve prolapse (MVP) represents the most frequent cause of primary mitral regurgitation (MR) in Western countries, with prevalence estimates of 0.6–3.1% [1,2]. The condition was first systematically described by Barlow and colleagues in 1963 [3]. Historically, MVP pathophysiology was interpreted primarily as a consequence of valvular incompetence and chronic volume overload, with generally favorable prognosis in the absence of significant MR or left ventricular (LV) systolic remodeling or dysfunction [1]. In this framework, the degree of LV enlargement was presumed to correlate directly with MR severity, in accordance with established principles of chronic volume overload states [4]. However, clinicians and investigators repeatedly observed a puzzling discordance: patients with Barlow disease, characterized by myxomatous degeneration with bileaflet prolapse, frequently exhibited disproportionate LV enlargement that exceeded predictions based on quantified transvalvular regurgitant volume [5]. This observation led to the “Barlow cardiomyopathy” hypothesis, proposing an intrinsic myocardial disease process independent of hemodynamic burden [6]. The advent of cardiovascular magnetic resonance (CMR) imaging has substantially refined the understanding of MVP pathophysiology and challenged this paradigm. El-Tallawi and colleagues in 2021 introduced the concept of doming volume (DV), also expressed as prolapse volume as a previously unquantified hemodynamic parameter contributing to total LV volume load [7]. Doming volume refers to the systolic volume displaced into the left atrium by prolapsing mitral leaflets, which is commonly not captured by conventional echocardiographic volumetric assessments. This concept has profound implications for understanding MVP pathophysiology, MR quantification, and surgical decision-making.

## 2. Pathophysiology of Mitral Valve Prolapse: Beyond Mitral Regurgitation

Structural abnormalities. From an anatomopathological perspective, there are two main types of mitral valve prolapse: myxomatous MVP, also known as Barlow’s disease, and MVP related to fibroelastic deficiency [2]. Myxomatous degeneration represents the histopathological hallmark of primary MVP and is the variant typically associated with greater DV. It is characterized by proteoglycan accumulation within the spongiosa layer of valve leaflets and degradation of organized collagen and elastin fibers [8]. This process results in leaflet thickening, redundancy, and loss of structural integrity. In Barlow disease, the classic form of myxomatous MVP, extensive leaflet involvement typically affects multiple segments of both leaflets, producing marked billowing and systolic displacement into the left atrium [9]. These structural abnormalities create an anatomical substrate for DV formation during systolic contraction.

Myocardial involvement and fibrosis patterns. Beyond valvular pathology, myocardial involvement in MVP has been increasingly recognized through advanced CMR tissue characterization. Late gadolinium enhancement (LGE) studies demonstrate replacement fibrosis in approximately 20–25% of MVP patients, predominantly localized to the basal and mid-inferolateral wall and papillary muscles [10,11]. This fibrosis pattern correlates with ventricular arrhythmias and may reflect chronic mechanical stress from abnormal papillary muscle traction and leaflet-ventricular interactions [12]. T1 mapping and extracellular volume quantification can detect diffuse interstitial fibrosis even in the absence of focal LGE, suggesting a spectrum of myocardial involvement that extends beyond overt replacement fibrosis [13,14,15].

Mitral Annular Disjunction. Mitral annular disjunction (MAD), defined as atrial displacement of the posterior mitral leaflet hinge point from the ventricular myocardium, is present in 30–50% of MVP patients and represents an important anatomical substrate for arrhythmic risk [16,17,18]. MAD creates abnormal systolic curling motion of the basal inferolateral wall and may contribute to mechanical stress, fibrosis development, and ventricular ectopy [19]. Recent studies distinguish true MAD, characterized by persistent annular–myocardial separation throughout both systole and diastole, from pseudo-mitral annular disjunction, which is apparent exclusively during systole, with important implications for risk stratification [20].

### The Concept of Doming Volume

**Figure 1:**
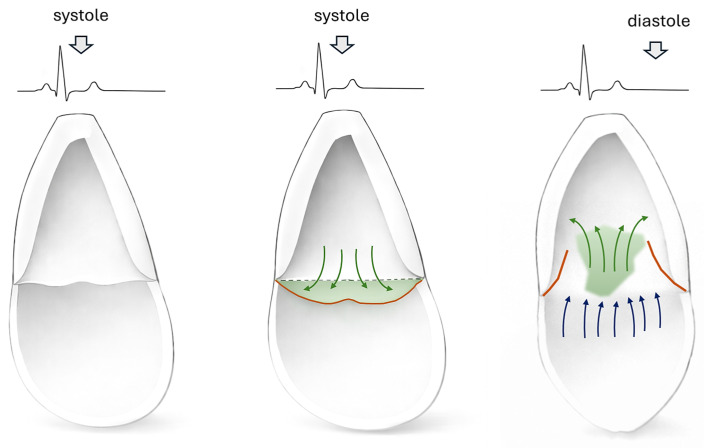
Conceptual representation of doming volume. Left panel: Systolic configuration in normal mitral valve with proper leaflet coaptation at the level of the mitral annular plane and absence of doming volume. Middle panel: Systole in mitral valve prolapse, showing displacement of the mitral leaflets into the left atrium and formation of the doming volume (green arrows, green area). Right panel: During diastole, in addition to the physiological transmitral inflow from the left atrium (blue arrows), the doming volume re-enters the left ventricle (green arrows, green area). Beyond this schematic representation, DV represents a three-dimensional blood compartment formed during systole, whose size and geometry depend on mitral valve morphology, and the severity and extent of leaflet prolapse.

DV, also termed prolapse volume, represents a three-dimensional blood compartment formed at end-systole between the mitral annular plane and the ventricular surface of prolapsing mitral leaflets [7,21,22,23]. DV remains trapped beneath the prolapsing leaflets on the atrial aspect of the atrioventricular groove but does not traverse the valve plane [22,23] (Figure 1). The hemodynamic significance of DV derives from its mechanical coupling to ventricular contraction. During systole, the LV myocardium must generate force to displace not only the LV stroke volume (LVSV) ejected into the aorta and the regurgitant volume propelled into the left atrium, but also the DV that balloons into the atrial space while remaining connected to the ventricle through the prolapsing leaflet tissue [7]. This creates an additional volume load that contributes to LV wall stress and chamber remodeling yet is invisible to conventional echocardiographic volumetric and flow-based MR quantification methods [21,24]. The most validated method for DV quantification employs CMR cine imaging with systematic measurement in three standard long-axis views [7,21].

**Figure 2:**
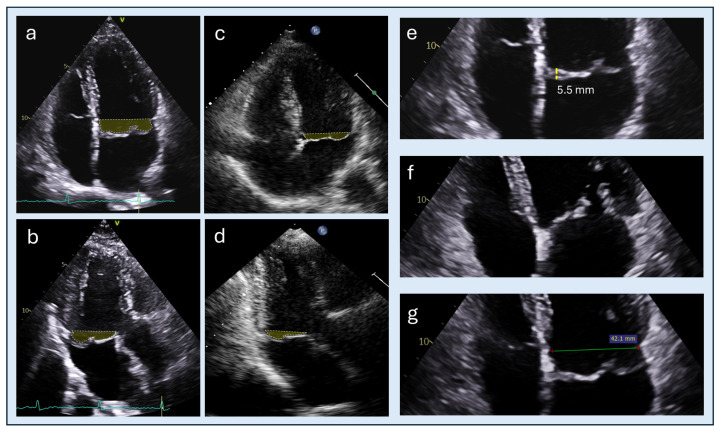
Determinants of doming volume magnitude. Left panels: Myxomatous (Barlow-type) mitral valve prolapse shown in the four-chamber (**a**) and three-chamber (**b**) views, characterized by thickened, redundant leaflets, bileaflet involvement, and annular dilatation, compared with fibroelastic deficiency in the four-chamber (**c**) and three-chamber (**d**) views, typically showing thin leaflets, localized prolapse, and relatively limited annular enlargement. Right panels: Main anatomical determinants of doming volume magnitude, including leaflet thickness > 5 mm (**e**), leaflet redundancy (**f**), and mitral annular diameter > 35 mm (**g**). Doming volume is highlighted in green.

Determinants of DV Magnitude. The magnitude of DV varies substantially across MVP phenotypes. A key factor is represented by the number of leaflets involved and the extent of leaflet redundancy. In the landmark El-Tallawi cohort of 157 patients, DV was significantly larger in bileaflet prolapse compared to single-leaflet prolapse: median 15.7 mL (interquartile range 11.3–20.6 mL) vs. 3.3 mL (IQR 1.2–5.5 mL), *p* < 0.001 [7]. In a study involving 81 MVP patients, Levy and colleagues found that patients with myxomatous (Barlow-type) MVP had significantly larger prolapse volumes compared to fibroelastic disease (11 ± 9 mL vs. 2 ± 2 mL, *p* < 0.001) [21]; on note, multivariable analysis identified bileaflet involvement, posterior leaflet thickness > 5 mm, and annular diameter > 35 mm as independent predictors of prolapse volume > 10 mL [21]. Another element may be represented by the MAD. Beyond its established association with arrhythmic risk, MAD may also have important implications in the context of DV. The abnormal systolic displacement of the annulus and exaggerated basal wall motion may amplify leaflet excursion and contribute to increased DV, thereby augmenting total left ventricular volume load and mechanical stress. A direct quantitative relationship between MAD and DV has not been systematically investigated and remains speculative, being primarily based on pathophysiological considerations; nevertheless, their frequent coexistence suggests a potential synergistic effect on ventricular remodeling and arrhythmic substrate, warranting further investigation. Table 1 and Figure 2 show a summary of the main anatomical and morphological parameters associated with doming volume magnitude in mitral valve prolapse.

## 3. Imaging Assessment

### 3.1. Echocardiography

**Figure 3:**
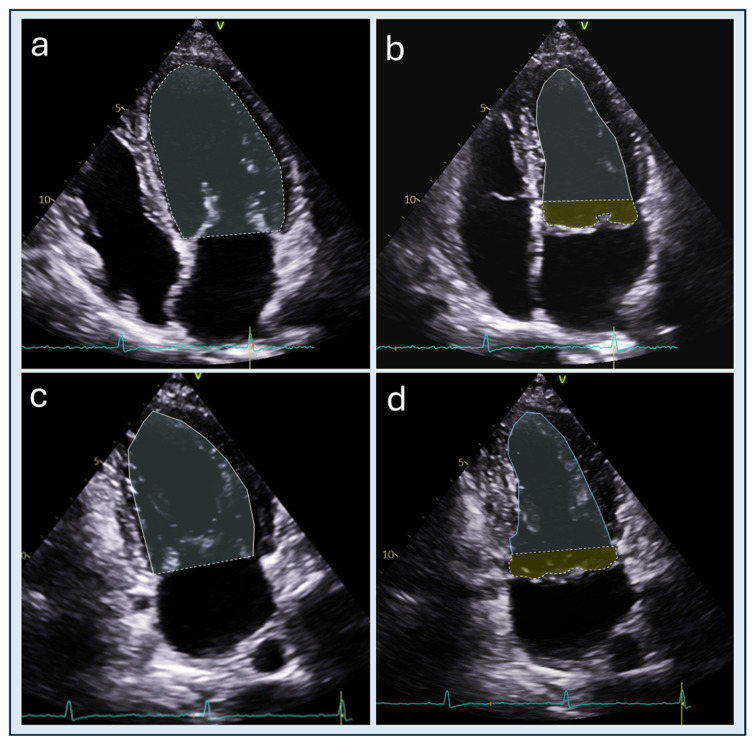
Apical four-chamber and two-chamber views used for biplane volumetric assessment of left ventricular volumes and ejection fraction according to the modified Simpson’s rule. Four-chamber view at end-diastole (**a**) and at end-systole (**b**), two-chamber view at end-diastole (**c**), and at end-systole (**d**). Endocardial contours are traced excluding the region located above the mitral annular plane. In the presence of mitral valve prolapse, the blood compartment interposed between the mitral annular plane and the ventricular surface of the prolapsing leaflets (doming volume; highlighted in green) is generally excluded from end-systolic contouring, which may lead to underestimation of LV end-systolic volume and consequent overestimation of ejection fraction.

Transthoracic echocardiography (TTE) represents the first-line imaging modality for the diagnosis and longitudinal follow-up of MVP. In most patients, TTE allows accurate identification of prolapsing segments, characterization of leaflet thickness and redundancy, and grading of MR using an integrated multiparametric approach in accordance with current recommendations [25,26]. TTE is also the routinely used modality for serial evaluation of LV volumes and systolic function. According to current echocardiographic guidelines, quantitative assessment of LV ejection fraction (LVEF) should be routinely performed using the biplane method of disks (modified Simpson’s rule) based on two-dimensional endocardial contouring in apical four- and two-chamber views [25]. This approach, while still relying on simplified geometric assumptions, represents the recommended first-line method for left ventricular volumetric and functional evaluation in clinical practice. In the presence of MVP, however, an important geometric limitation emerges. The blood compartment located between the mitral annular plane and the ventricular surface of prolapsing leaflets, the DV, is commonly excluded from echocardiographic end-systolic cavity contouring, which may result in underestimation of LV end-systolic volume (Figure 3). This geometric exclusion may lead to overestimation of LVEF and SV and, when volumetric methods are applied to quantify MR, to underestimation of regurgitant burden, specifically when it comes to quantization of regurgitant fraction. Importantly, these effects are independent of intrinsic myocardial contractility and reflect a methodological limitation rather than preserved or supranormal systolic performance [7,21,22]. However, the impact of this limitation is not uniform across all patients and is more pronounced in specific scenarios, including bileaflet prolapse, complex or multiple regurgitant jets, and advanced myxomatous disease, where doming volume is larger and leaflet displacement is more extensive. The impact of DV on echocardiographic measurements provides a mechanistic explanation for the frequent discordance observed between echocardiography-derived and CMR-derived assessments of LV volumes, systolic function, and MR severity [27]. In contrast, CMR assessment of ventricular volumes and function is not based on geometric assumptions, as it evaluates the ventricles in their entirety through contiguous short-axis stacks [23]. This approach allows comprehensive volumetric analysis, ensuring that all portions of ventricular volume, including the doming volume, are incorporated into the calculation. This discordance is particularly pronounced in bileaflet prolapse and Barlow disease, where extensive leaflet redundancy and multisegment involvement generate larger doming volumes and greater systolic leaflet displacement [21,27,28]. In this setting, TTE may suggest preserved or supranormal LV systolic function despite advanced chamber remodeling, creating apparent inconsistencies between functional indices and structural findings. Direct echocardiographic quantification of doming volume is not methodologically feasible. Unlike the left ventricle, whose geometry can be reasonably approximated using standardized assumptions, the anatomical substrate defining doming volume is intrinsically irregular, highly patient-specific, and determined by the complex three-dimensional interaction of leaflet prolapse, chordal elongation, and annular deformation. Furthermore, prolapsing leaflet anatomy is not consistently captured within standard apical imaging planes, particularly in the presence of asymmetric or bileaflet disease, precluding reproducible delineation of the doming compartment [21,22]. Advanced echocardiographic techniques, including three-dimensional and transesophageal echocardiography, may provide more detailed and potentially more accurate visualization of MV anatomy and leaflet prolapse compared with standard TTE imaging. However, direct and reproducible quantification of DV remains challenging due to the complex, irregular, and highly patient-specific geometry of the doming compartment, as well as the lack of standardized acquisition and post-processing methods. In addition, these techniques are not routinely used for quantitative volumetric assessment in clinical practice and are typically reserved for selected cases. Importantly, to date, no studies have validated the quantification of DV using echocardiographic approaches, including three-dimensional or transesophageal imaging, nor have comparisons with reference methods such as CMR been systematically performed. Accordingly, dedicated validation studies are required before these techniques can be considered reliable for doming volume assessment. Despite these limitations, echocardiography remains indispensable for anatomical characterization of the mitral valve apparatus, evaluation of leaflet morphology and motion, and integrated assessment of MR severity. However, in patients with advanced myxomatous MVP, particularly those with bileaflet prolapse, echocardiographic volumetric measurements should be interpreted in the context of leaflet geometry and the potential exclusion of doming volume. Apparent preservation or supranormal values of LVEF and stroke volume should not be assumed to reflect normal myocardial performance when LV or left atrial remodeling is present or when imaging findings appear discordant with the clinical picture. In such situations, echocardiography provides essential anatomical and hemodynamic information; however, complementary imaging with CMR may be required when the grading of MR does not adequately correlate with the degree of cardiac remodeling or with the patient’s clinical status. In these cases, CMR allows a more accurate and physiologically consistent assessment of ventricular volumes and regurgitant burden [7,21].

### 3.2. Cardiovascular Magnetic Resonance Imaging

**Figure 4:**
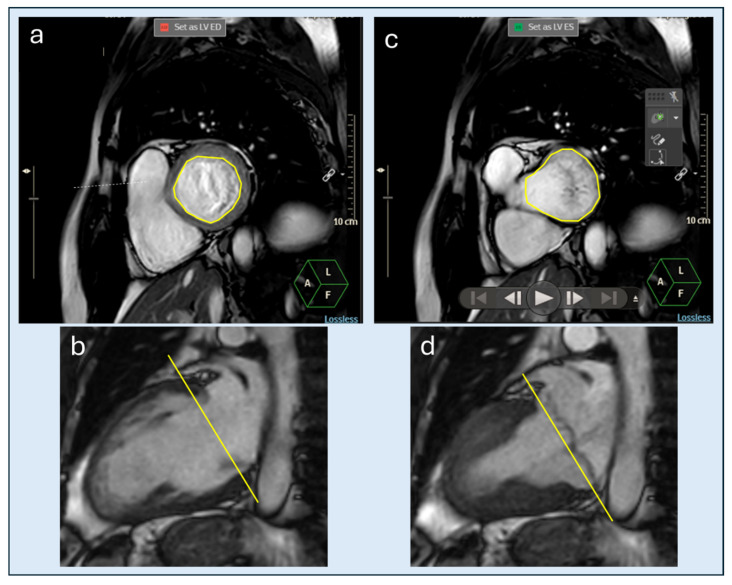
Cardiac magnetic resonance assessment of left ventricular volumes and ejection fraction with inclusion of doming volume. Representative cardiac magnetic resonance images illustrating left ventricular volumetric assessment. Basal short-axis view at end-diastole with left ventricular endocardial contouring (**a**) and corresponding long-axis reference view indicating the position of the basal slice (yellow line) (**b**). Basal short-axis view at end-systole (**c**) with corresponding long-axis reference view at end-systole (yellow line) (**d**). Side-by-side comparison with long-axis views enables accurate identification of the true ventricular extent and inclusion of the blood compartment located between the mitral annular plane and the ventricular surface of prolapsing leaflets into left ventricular volumetric analysis.

CMR has emerged as the reference standard for comprehensive MVP assessment, offering superior accuracy for chamber quantification, MR quantification, tissue characterization, and DV measurement [29,30]. Balanced steady-state free precession cine imaging provides a high temporal and spatial resolution assessment of valve morphology and ventricular function. Standard long-axis views (four-, three-chamber, two-chamber) and a short-axis stack covering the LV from base to apex are acquired [31]. Specifically, left ventricular volume and function assessment is based on the inclusion of the entire ventricle, verified by side-by-side long-axis views. This approach allows correct incorporation of the DV into volumetric analysis [23] (Figure 4).

**Figure 5:**
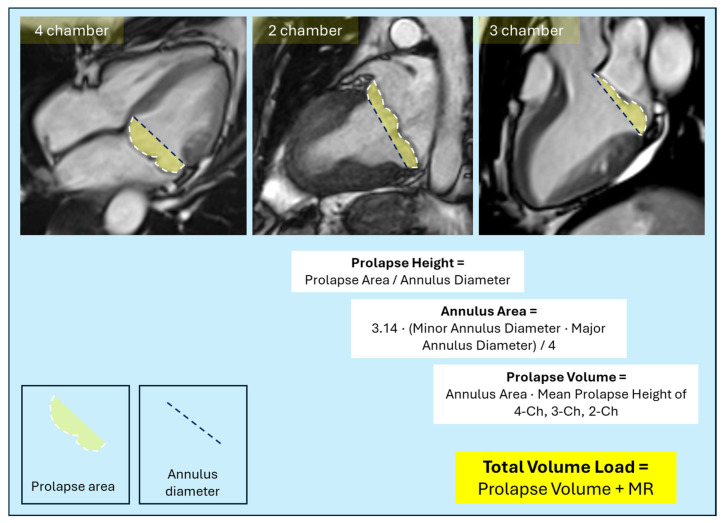
Cardiovascular magnetic resonance-based quantification of doming volume in mitral valve prolapse. Doming volume quantification using standard long-axis cine cardiovascular magnetic resonance images acquired at end-systole. Prolapse area is delineated in the four-chamber, two-chamber, and three-chamber views as the region bounded by the mitral annular plane and the ventricular surface of the prolapsing leaflets (green areas). The mitral annular plane is defined by a reference line connecting the medial and lateral annular hinge points. For each view, prolapse height is calculated as the ratio between prolapse area and annular diameter. Mean prolapse height is obtained by averaging measurements from the four-, three-, and two-chamber views. Mitral annular area is estimated assuming an elliptical geometry, using the three-chamber annular diameter as the minor axis and the larger of the four- or two-chamber diameters as the major axis. Doming volume is calculated as the product of mean prolapse height and annular area. The total volume load is defined as the sum of the doming volume and the mitral regurgitant volume.

In addition to preventing the exclusion of DV, a major strength of CMR is its ability to directly and quantitatively assess DV. The most rigorously validated method for DV quantification was established by El-Tallawi and colleagues using CMR cine imaging in a cohort of 157 MVP patients [7]. The protocol employs standard long-axis cine views acquired during routine CMR examinations, making it clinically feasible without specialized sequences. CMR examinations are performed on 1.5 T or 3.0 T scanners using steady-state free-precession (SSFP) cine sequences. Key technical parameters include: flip angle 65–85°, repetition time 3.0 ms, echo time 1.3 ms, in-plane spatial resolution 1.7–2.0 × 1.4–1.6 mm, slice thickness 6 mm with 4 mm interslice gap, and temporal resolution 35–40 ms [7]. Three standard long-axis views are acquired: four-chamber, three-chamber, and two-chamber. Analysis is performed at end-systole, defined as the phase with the smallest LV cavity area. This geometric approach assumes the DV approximates a truncated cone or ellipsoid, with the annular plane as the base and the mean prolapse height representing the perpendicular extent. Once the doming volume has been quantified, the total volume load on the left ventricle can be calculated as the sum of the regurgitant volume and the doming volume. On each of the three long-axis views, the stepwise CMR-based methodology used for doming volume quantification is summarized in Table 2 and shown in Figure 5. Validation against three-dimensional volumetric segmentation has demonstrated excellent correlation (r = 0.91, *p* < 0.001) [7]. Independent validation of the DV concept has been performed using four-dimensional flow (4D flow) CMR as a reference standard. Li and colleagues compared LVSV calculations with and without inclusion of DV against aortic forward flow measured by 4D flow CMR in 15 MVP patients [22]. When DV was excluded from LVSV calculation using the standard formula: LVSV = LVEDV − LV end-systolic volume, the correlation with 4D flow-derived aortic forward flow was moderate (intraclass correlation coefficient ICC = 0.75). However, when DV was added to the standard LVSV calculation using the formula LVSV_MVP = LVEDV − (LVESV + DV), the correlation with 4D flow improved substantially (ICC = 0.86) [22]. This improvement in agreement supports the physiological validity of including DV in total LVSV assessment. The DV in this validation cohort was 11.2 ± 8.7 mL, with individual values ranging from 2 to 31 mL [22]. Importantly, when DV was not accounted for, LVEF was overestimated (61 ± 14% vs. 52 ± 11%, *p* < 0.001), because the denominator represented by LVEDV did not include the DV that is mechanically coupled to the ventricle [22]. In the original validation, inter-observer ICC was 0.89 (95% CI 0.84–0.93) and intra-observer ICC was 0.92 (95% CI 0.88–0.95), indicating excellent reproducibility [7]. The method requires only standard long-axis cine images that are acquired in all CMR examinations and measurement time averaged 8–12 min per case, making the technique clinically feasible for routine CMR reporting [22,29].

### 3.3. Echocardiography vs. CMR Comparison

Multiple studies have documented discordance between echocardiography and CMR in MVP assessment [27,28]. The PROMIS-MR trial found that echocardiography reclassified MR severity in 43% of cases when compared to CMR as the reference standard [24]. As discussed above, echocardiographic exclusion of DV from end-systolic volume may lead to overestimation of LVEF, particularly in specific clinical scenarios. In the El-Tallawi cohort, when DV was included in CMR-derived LVESV calculations, mean LVEF decreased from 61 ± 14% to 52 ± 11% (*p* < 0.001) [7]. Levy and colleagues demonstrated that in MVP patients with DV > 14 mL, PISA-derived regurgitant volume underestimated CMR-quantified MR by 26 ± 32 mL [21]. This resulted in MR grade reclassification in 49% of bileaflet prolapse patients [7]. It should be acknowledged that some of these studies have methodological limitations that may influence the interpretation of echocardiography–CMR discordance, including relatively small sample sizes, particularly in patients with follow-up, limited use of transesophageal echocardiography without systematic correlation with CMR in discordant cases, and interobserver variability in mitral regurgitation assessment. Current guidelines recommend CMR for MR quantification when echocardiographic assessment is technically limited or discordant with clinical presentation [32,33]. In MVP with bileaflet prolapse, CMR should be considered even when echocardiographic image quality is adequate, given the limitations imposed by DV exclusion [23]. In clinical practice, TTE remains the first-line imaging modality, while transesophageal echocardiography represents the next step in cases of uncertainty or when detailed anatomical assessment is required, particularly in the pre-operative setting. CMR may be integrated into this workflow in cases of discordant findings, inconclusive echocardiographic assessment, or when a more accurate quantification of ventricular volumes and regurgitant burden is needed. Despite its strengths, the use of CMR may be limited by availability, cost, and patient- or technique-related constraints, and it should be considered complementary to echocardiography rather than a replacement in routine clinical practice. The main imaging implications of doming volume and the resulting differences between echocardiography and cardiovascular magnetic resonance are summarized in Table 3.

## 4. Clinical Implications

The clinical significance of DV has been validated in multiple cohorts demonstrating its impact on LV remodeling, MR quantification, and risk stratification. The landmark El-Tallawi study of 157 MVP patients established the fundamental relationship between DV and disproportionate LV enlargement [7]. Patients with bileaflet prolapse had significantly larger indexed LVEDV despite similar transvalvular MR severity. When DV was added to regurgitant volume to calculate total volume load, the correlation with indexed LVEDV improved substantially, explaining the previously puzzling discordance. Levy and colleagues validated these findings in an independent cohort, demonstrating that DV averaged 11 ± 9 mL in myxomatous MVP vs. 2 ± 2 mL in fibroelastic deficiency [21]. The threshold of DV > 14 mL identified patients with echocardiographic underestimation of hemodynamic burden. Li and colleagues further validated the concept using 4D flow CMR as a reference, showing that inclusion of DV in LVSV calculations improved agreement with directly measured aortic forward flow [22]. Manceau and colleagues identified a high SV phenotype in approximately 12% of MVP patients, characterized by indexed SV ≥ 65 mL/m^2^, disproportionate LV dilatation, and a higher prevalence of MAD (67.3% vs. 30–40% in other groups) [34]. This phenotype likely represents the extreme end of the DV spectrum, where the combined effects of large regurgitant volume and substantial DV produce marked volume overload. The clinical and prognostic implications associated with large doming volume in mitral valve prolapse are summarized in Table 4.

Correlation with Left Ventricular Remodeling. Longitudinal studies have demonstrated that LV remodeling in MVP can occur even with mild-to-moderate MR when DV is substantial [35]. Progressive LV dilatation over time correlates with total volume load and predicts adverse outcomes, including heart failure symptoms, atrial fibrillation, and need for surgical intervention [36], suggesting that disproportionate LV enlargement in bileaflet MVP reflects total volume load rather than intrinsic myocardial disease [7,13]. However, the relationship between volume load and remodeling is not purely linear. Pype and colleagues proposed a multifactorial model in which LV remodeling in MVP results from the combination of: (a) total volume overload (MR + DV), (b) chronic mechanical stress from abnormal valve-papillary-ventricular interactions, (c) genetic predisposition to connective tissue abnormalities that may affect both valve and myocardium, and (d) arrhythmia-mediated remodeling in patients with high ectopic burden [13]. This integrated model acknowledges that while DV explains much of the observed remodeling, additional factors contribute to the phenotypic heterogeneity of MVP.

Impact on Mitral Regurgitation Quantification. Accurate MR quantification is essential for clinical decision-making, yet DV may introduce inaccuracies in conventional assessment methods. Echocardiographic techniques including PISA, vena contracta, and volumetric methods all assume that LVSV minus aortic forward flow equals regurgitant volume [26]. This assumption is violated when substantial DV is present. In the Levy cohort, prolapse volume > 14 mL was associated with underestimation of regurgitant volume by the echocardiographic proximal isovelocity surface area (PISA) method compared to CMR phase-contrast quantification, with a mean bias of −26 ± 32 mL [21]. This degree of underestimation frequently results in MR grade misclassification, with potential implications for surgical timing. The 14 mL threshold also distinguished patients at risk for MR severity reclassification when total volume load was considered [7]. In fact, in the El-Tallawi study, 49% of bileaflet prolapse patients were reclassified to a higher MR grade when total volume load was considered [7]. CMR-based MR quantification using the difference between LV total SV including DV and phase-contrast aortic forward flow provides a more accurate assessment independent of DV [37]. This approach has been validated against multiple reference standards and demonstrates superior reproducibility compared to echocardiography [27]. Current guidelines acknowledge CMR as the reference standard for MR quantification when echocardiographic assessment is uncertain or discordant with clinical findings [32,33].

Implications for Surgical Timing. Current guidelines recommend mitral valve surgery for severe primary mitral regurgitation based on a combination of symptom status, markers of left ventricular remodeling or dysfunction, and the presence of adverse clinical features [31,32]. However, these thresholds were established using echocardiographic measurements that exclude DV. Recognition of DV raises important questions about optimal surgical timing in MVP. Patients with large DV may have greater total volume load than appreciated by conventional echocardiography, potentially warranting earlier intervention. Conversely, apparently “supranormal” LVEF values in the presence of large DV may reflect methodological artifact rather than true contractile reserve, suggesting that current LVEF thresholds may be too conservative. Tribouilloy and colleagues demonstrated that LV end-systolic dimension provides superior prognostic information compared to LVEF for predicting post-operative LV dysfunction in MVP [38], possibly because linear dimensions are less affected by DV exclusion than volumetric ejection fraction. Importantly, doming volume assessment does not replace current guideline criteria but provides a physiological framework to interpret their limitations in advanced myxomatous MVP. The integration of DV into surgical decision-making algorithms remains an area of active investigation.

Prognostic Considerations. Beyond its impact on volume load quantification, DV may have independent prognostic significance. The mechanical stress imposed by large DV may contribute to progressive annular dilatation and chordal elongation, accelerating disease progression. The relationship between DV and arrhythmic risk is an emerging area of interest. Large DV frequently coexists with MAD, and their combination may amplify mechanical stress on the basal inferolateral wall, promoting myocardial fibrosis and ventricular arrhythmias [11,14]. Abnormal myocardial mechanics, including exaggerated basal systolic curling and a characteristic double-peak strain pattern, have been shown to correlate with both fibrosis and arrhythmic events in MVP and are most pronounced in patients with bileaflet MVP and large DV [11]. Accordingly, comprehensive risk stratification in MVP increasingly relies on multimodality imaging integrating valve anatomy, hemodynamic burden including DV, myocardial tissue characterization, and strain analysis [29,39]. Machine learning analyses of multicenter CMR registries further support this integrated approach, identifying high-risk phenotypes characterized by bileaflet prolapse and large DV [40].

**Table 4:** Clinical and prognostic implications of doming volume in mitral valve prolapse.

Domain	Association with Large DV	Clinical Relevance	Potential Clinical Action	Reference
LV remodeling	Disproportionate LV dilatation	Explains LV enlargement	Consider total volume load (including DV) when interpreting LV enlargement; favor CMR in case of discordance	[7]
MR assessment	Echo underestimation	MR reclassification	Use CMR for accurate quantification when TTE findings are incongruent with clinical or structural data	[21]
Arrhythmic risk	DV + MAD + fibrosis	Higher arrhythmic burden	Consider comprehensive risk stratification including tissue characterization and rhythm monitoring in patients with large DV	[11]
Risk stratification	High-risk phenotype	Integrated imaging needed	Adopt multimodality imaging approach combining valve anatomy, ventricular volumes, DV, and myocardial tissue characterization	[40]

DV, doming volume; LV, left ventricle; CMR, cardiac magnetic resonance; MAD, mitral annular disjunction; MR, mitral regurgitation.

## 5. Current Limitations

Despite increasing interest, the current evidence on DV in MVP has several limitations. There is relevant variability in measurement methods, as CMR allows direct quantification but lacks fully standardized protocols across centers, while TTE does not permit direct measurement and relies only on non-validated indirect surrogates.

Available studies also show significant heterogeneity between cohorts, including differences in valve phenotype, extent of leaflet involvement, severity of MR, and prevalence of MAD, which limits the comparability and generalizability of results. In addition, most data derive from small, single-center, and predominantly retrospective studies, without large prospective validation. Another important limitation is the lack of standardized thresholds to define clinically relevant DV, as proposed cut-offs are based on limited datasets and have not been validated in outcome-driven studies. Finally, the association between doming volume and clinical outcomes, particularly ventricular arrhythmias, remains uncertain in terms of causality, as doming volume frequently coexists with other high-risk features and may represent a marker of disease severity rather than an independent determinant of adverse events.

## 6. Future Perspectives and Research Directions

Recognition of DV as a key component of volume load in MVP opens important avenues for future research. Prospective longitudinal studies are needed to define the evolution of DV and its relationship with LV remodeling, MR progression, and clinical outcomes. A major unmet need is the identification of clinically relevant thresholds. Key questions include: What is the optimal DV cut-off associated with adverse remodeling or indication for intervention? And, should DV be indexed to BSA? Development of DV-adjusted thresholds for LV dimensions and function may help refine surgical timing. The role of DV in surgical decision-making also requires clarification, including its ability to predict post-operative LV reverse remodeling, persistent LV dilatation, and repair durability. Advanced imaging techniques, particularly 4D flow, together with strain analysis and parametric mapping, may improve understanding of the biomechanical impact of DV and its associated flow patterns and energy loss [41]. The interplay between DV, MAD, myocardial fibrosis, and ventricular arrhythmias warrants evaluation in large prospective cohorts. Whether DV independently contributes to arrhythmic risk or reflects disease severity remains uncertain. Finally, integration of DV into multiparametric risk models, including sudden cardiac death prediction, and the use of artificial intelligence-based phenotyping may improve identification of high-risk phenotypes [42,43]. Current guideline thresholds are based on echocardiographic metrics that exclude DV, potentially leading to underestimation of disease severity, supporting future investigation aimed at validating the incorporation of DV assessment, particularly in bileaflet MVP or discordant imaging findings.

## 7. Conclusions

Recognition and quantification of DV represent a paradigm shift in MVP pathophysiology and hemodynamic assessment. DV contributes to total LV volume load independently of transvalvular MR and explains the longstanding observation of disproportionate LV enlargement in bileaflet MVP and Barlow disease. Exclusion of DV from echocardiographic volumetric assessment underlies many apparent inconsistencies between functional indices and structural remodeling in MVP, including overestimation of LVEF and underestimation of regurgitant burden. These discrepancies reflect methodological limitations rather than preserved or supranormal myocardial contractile function. CMR has emerged as the reference standard for comprehensive MVP evaluation, enabling accurate quantification of LV volumes, MR severity, and DV, while providing complementary prognostic information through myocardial tissue characterization. In advanced myxomatous MVP, particularly with bileaflet involvement, consideration of DV may inform interpretation of LV function, remodeling, and MR severity and may help refine timing of intervention in patients with discordant imaging findings, although further evidence is required before DV assessment can be translated into operative clinical indications or decision-making thresholds.

## Figures and Tables

**Table 1:** Determinants of doming volume magnitude in mitral valve prolapse.

Parameter	Description	Reference
Leaflet involvement	Bileaflet vs. single-leaflet prolapse	[7]
Valve phenotype	Myxomatous (Barlow-type) vs. fibroelastic disease	[21]
Independent predictors of DV > 10 mL	Bileaflet prolapse; posterior leaflet thickness > 5 mm; annular diameter > 35 mm	[21], multivariable analysis

**Table 2:** CMR-based methodology for doming volume quantification. Stepwise description of the geometric approach used to quantify doming volume from standard long-axis cardiovascular magnetic resonance cine images. Measurements are performed at end-systole and integrate annular dimensions and leaflet prolapse geometry to derive doming volume.

Step	Measurement	Description
1	Annular diameter measurement	Reference line connecting medial and lateral mitral annular hinge points, defining the annular plane.
2	Prolapse area delineation	Area bounded by the annular plane and ventricular surface of prolapsing leaflets, representing the 2D projection of DV.
3	Prolapse height calculation	Calculated as prolapse area divided by annular diameter (height = area ÷ diameter).
4	Mean prolapse height	Average of prolapse heights from four-, three-, and two-chamber views.
5	Annular area determination	Calculated using an elliptical formula, with the three-chamber diameter as minor axis and the larger of four- or two-chamber diameters as major axis.
6	Doming volume calculation	DV calculated as mean prolapse height multiplied by annular area (DV = mean height × annular area).

**Table 3:** Imaging implications of doming volume in mitral valve prolapse. Comparison between echocardiography and cardiovascular magnetic resonance assessment in the presence of doming volume, highlighting methodological limitations, corrected measurements, and clinical impact, with corresponding references.

Parameter	TTE	CMR	Impact	Reference
LVESV	May be underestimated	Accurately measured	LVEF overestimation	[7]
LVEF	May be overestimated	Lower when DV included	Pseudo-hyperdynamic LV	[7]
LVSV	May be overestimated	Corrected incl. DV	Misclassification of load	[19]
MR quantification	May be underestimated	Accurate (SV–Ao flow)	MR grade reclassification	[18]
DV assessment	Generally not feasible	Directly quantifiable	May explain LV remodeling	[7]

Ao, aorta; TTE, transthoracic echocardiography; CMR, cardiovascular magnetic resonance; LVESV, left ventricular end-systolic volume; LVEF, left ventricular ejection fraction; LVSV, left ventricular stroke volume; DV, doming volume; EF, ejection fraction; LV, left ventricle; MR, mitral regurgitation; SV, stroke volume.

## Data Availability

The examinations from which the images were taken are always available on the portal of the institute to which the corresponding author belongs.

## Abbreviations

The following abbreviations are used in this manuscript:

MVP	Mitral valve prolapse
MR	Mitral regurgitation
LV	Left ventricular
CMR	Cardiovascular magnetic resonance
DV	Doming volume
LGE	Late gadolinium enhancement
MAD	Mitral annular disjunction
TTE	Transthoracic echocardiography
LVEF	Left ventricular ejection fraction
LVEDV	Left ventricular end-diastolic volume
LVESV	Left ventricular end-systolic volume
LVSV	Left ventricular stroke volume
SSFP	Steady-state free precession
ICC	Intraclass correlation coefficient
PISA	Proximal isovelocity surface area
Ao	Aorta
SV	Stroke volume
EF	Ejection fraction
